# Cardiac resynchronization therapy defibrillator: *de novo* implant in a COVID-19 patient by an echo-guided axillary venous approach

**DOI:** 10.2217/fca-2021-0055

**Published:** 2022-06-23

**Authors:** Aldo Agea, Maria C Bellino, Domenico Gianfrancesco, Francesco Palma, Giuseppe A Carpagnano, Rita MR Torraco, Maria M Marino, Paola Persichella, Sebastiano Zingaro, Fabio Siena, Roberto Di Tullio, Giovanni Deluca

**Affiliations:** ^1^Monsignor Dimiccoli Hospital, Cardiology, Barletta, Puglia, 76121, Italy; ^2^Ospedale Lorenzo Bonomo, Cardiology, Andria, Puglia, 76123, Italy

**Keywords:** atrioventricular block, COVID-19, cardiac resynchronization therapy, defibrillators, device implantation, echo-guided venous approach, infection, myocardial infarction, TYRX

## Abstract

The COVID-19 pandemic has seriously revolutionized the management of patients who need an implanted cardiac implantable electronic device. We report, for the first time, a successful cardiac resynchronization therapy defibrillator implantation procedure in an 82-year-old man affected by COVID-19, recent myocardial infarction, second-degree 2:1 atrioventricular block and left bundle branch block.

COVID-19 is an infectious disease caused by a novel coronavirus that was discovered in December 2019. Most people infected will experience fever, cough, fatigue, myalgia, diarrhea, vomiting and atypical pneumonia, with potential cardiac complications such as pulmonary embolism, pericarditis and myocarditis [1]. The pandemic that resulted from the spread of the virus has revolutionized healthcare systems and patient care, making it more challenging. The implantation of defibrillators, a life-saving treatment, has been often postponed until the recovery from the infection due to the greater difficulty of the procedure in these patients [2]. In this article we present a successful procedure of cardiac resynchronization therapy defibrillator (CRT-D) implantation in a COVID-19 patient. CRT-D is a device used in patients affected by heart failure with reduced ejection fraction and specific electrocardiographic abnormalities. The device used for CRT-D consists of three leads that connect the defibrillator to the right upper chamber of the heart (right atrium) and both lower chambers (right and left ventricles). The leads are essential for biventricular pacing. The device improves cardiac function and symptoms and reduces morbidity and mortality in appropriately selected group of patients [3].

## Presentation of case

### Patient history & initial assessment

**Figure 1.**
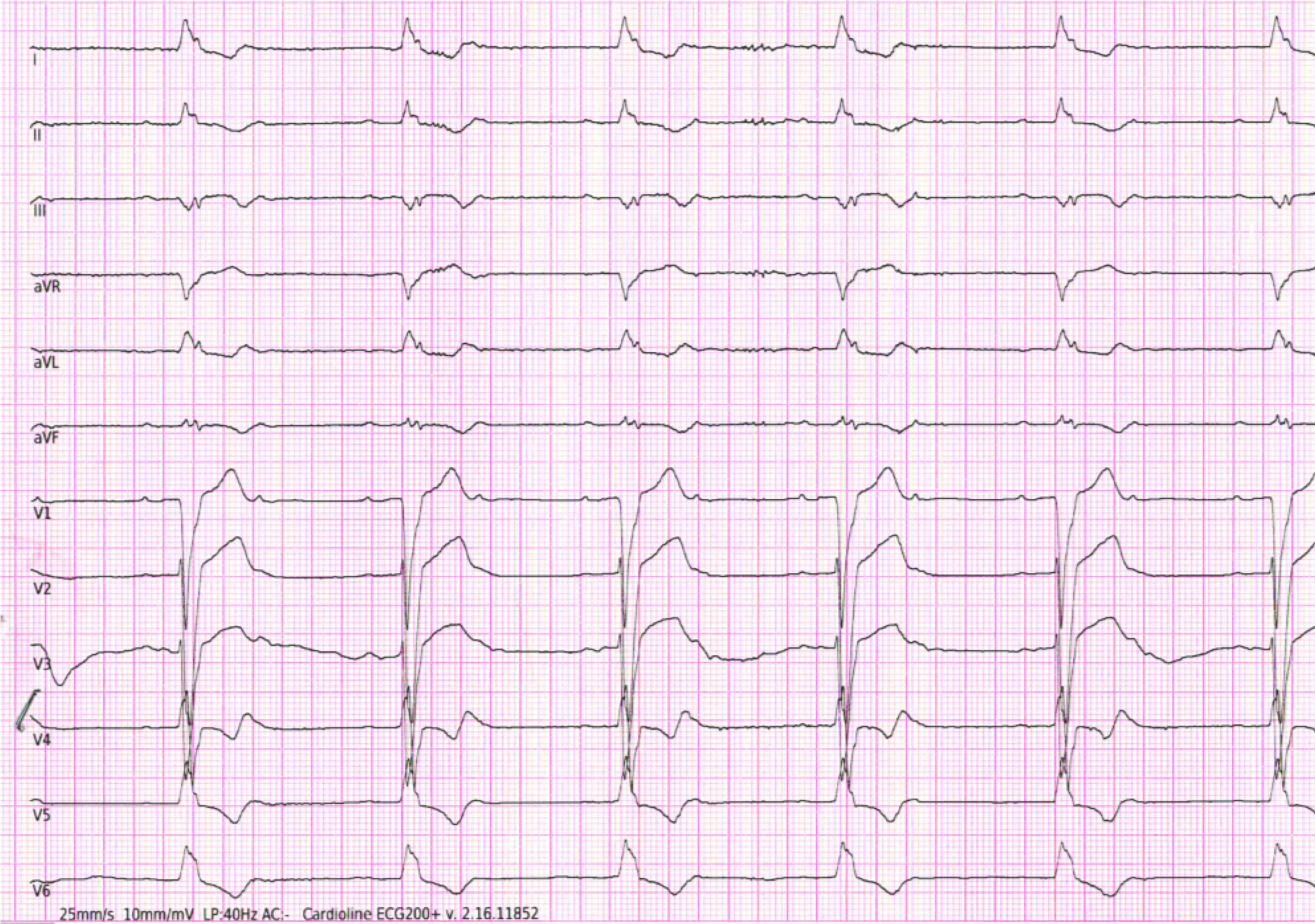
Twelve-lead ECG revealed sinus rhythm, second-degree 2:1 atrioventricular block with ventricular rate at 40 beats/min and left bundle branch block.

An 82-year-old man affected by hypertension and diabetes was admitted to our ward in February 2021 because of chest pain and dyspnea. ECG revealed a new onset of left bundle branch block (LBBB). A diagnosis of ST elevation myocardial infarction (MI) was made, and the patient was promptly treated with primary percutaneous coronary intervention on three major vessels (left main, circumflex artery and right coronary artery). Echocardiogram showed a severe left ventricular (LV) dysfunction with an ejection fraction (EF) of 20% calculated with the Simpson biplane method. During the hospitalization, he tested positive for SARS-CoV-2 via nasopharyngeal swab. Subsequent high-resolution computed tomography did not show lung involvement of the infection. Laboratory analysis showed a mild lymphopenia and slight increase of C-reactive protein. Peripheral O_2_ saturation was 98% with 4 l/min O_2_ therapy. COVID-19 standard therapy with azithromycin 500 mg/day, enoxaparin 4000 UI/day, remdesivir and empirical treatment with ceftriaxone 2 g/day was started to avoid potential cardiac and respiratory complications of the infection, such as respiratory failure, pulmonary embolism, myocarditis and arrhythmias. Due to his clinically stable condition, the patient was discharged in optimal medical therapy from our ward and transferred to a COVID rehabilitative hospital. Given the severe LV dysfunction, the patient underwent repeat echocardiography 40 days after the MI to assess the potential need for primary prevention using an implantable cardioverter defibrillator. The patient was urgently transferred to our ward 3 days later because of symptomatic bradycardia at 40 beats/min. ECG documented a sinus rhythm, second-degree 2:1 atrioventricular (AV) block and complete LBBB (Figure 1). Infusion of isoprenaline was started immediately, with rapid increase of the ventricular rate. Nasopharyngeal swab for SARS-CoV-2 remained positive for the infection. Therapy against COVID-19 was continued, and 98% peripheral O_2_ saturation was achieved with 4 l/min of oxygen. Severe reduction of EF was still detected on the echocardiogram. For the following days the patient was treated with continuous isoprenaline infusion. The 24-h ECG monitoring documented frequent non-sustained ventricular tachycardia episodes; nevertheless, we were unable to suspend the infusion of isoprenaline due to the decreased ventricular rate. Afterward, a new nasopharyngeal swab was performed and was positive for SARS-CoV-2.

### Management

**Figure 2.**
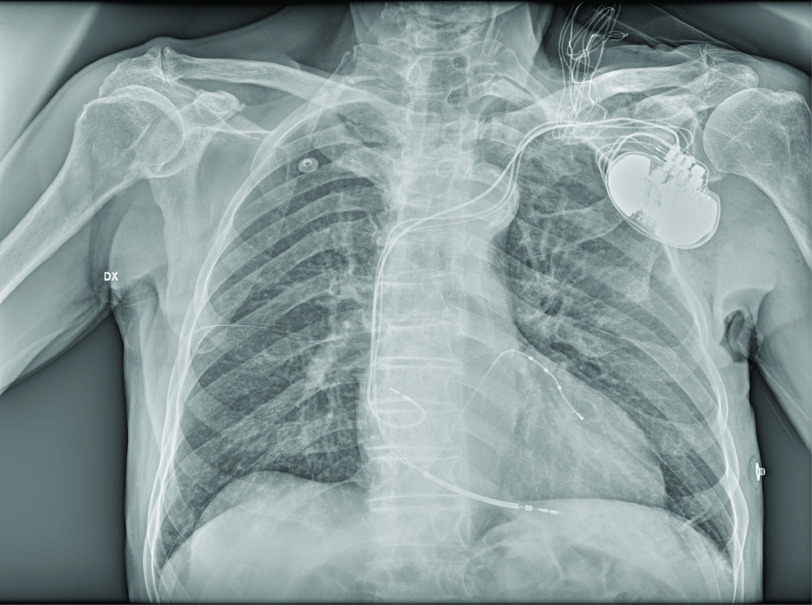
Bedside chest x-ray showing the cardiac resynchronization therapy defibrillator (Biotronik Rivacor 7 HF).

**Figure 3.**
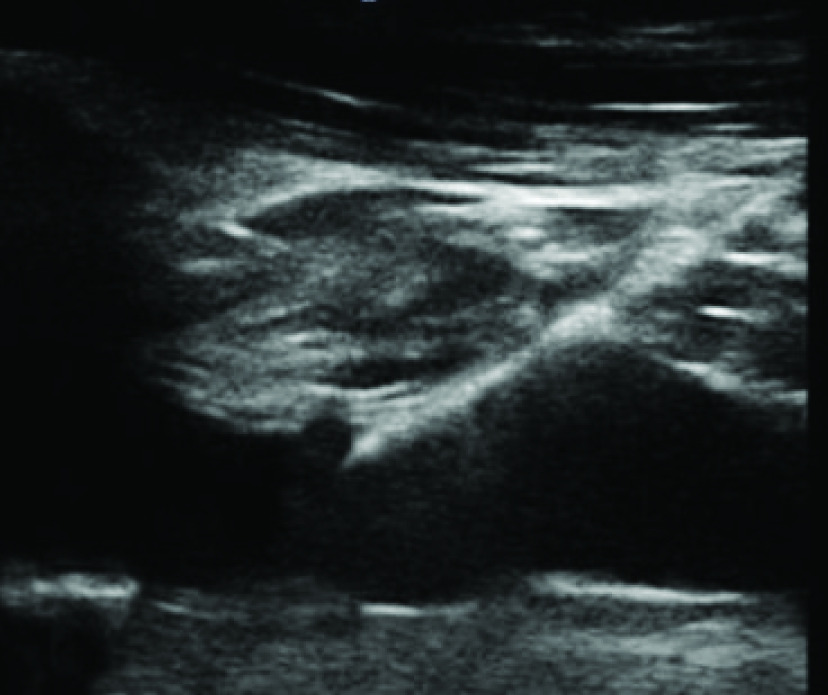
The needle inside the left axillary vein.

Given the persistent 2:1 AV block without therapy, the complete LBBB and the severe LV dysfunction, we decided to proceed to CRT-D implantation despite the patient’s ongoing SARS-CoV-2 infection. The procedure was performed in a negative-pressure operating room which was routinely disinfected, and by a limited number of healthcare professionals: two operators, two nurses and one radiologist at the console. All of them wore level III personal protective equipment: disposable surgical cup, medical protection mask (FFP3), external surgical mask, goggles, full-face respiratory protective device, double gloves, work uniform and leaded gown. A sterile coat and gloves were also worn. A CRT-D (Rivacor 7HF; Biotronik, Berlin, Germany) was implanted without complications (Figure 2). Atrial passive fixation, ventricular active fixation and multipolar coronary sinus LV leads were positioned by triple left axillary ultrasound-guided puncture, a method that we routinely use in our department (Figure 3) [4]. The device was placed in the pocket with an antimicrobial envelope (TYRX™, Medtronic, MN, USA).

### Outcome

**Figure 4.**
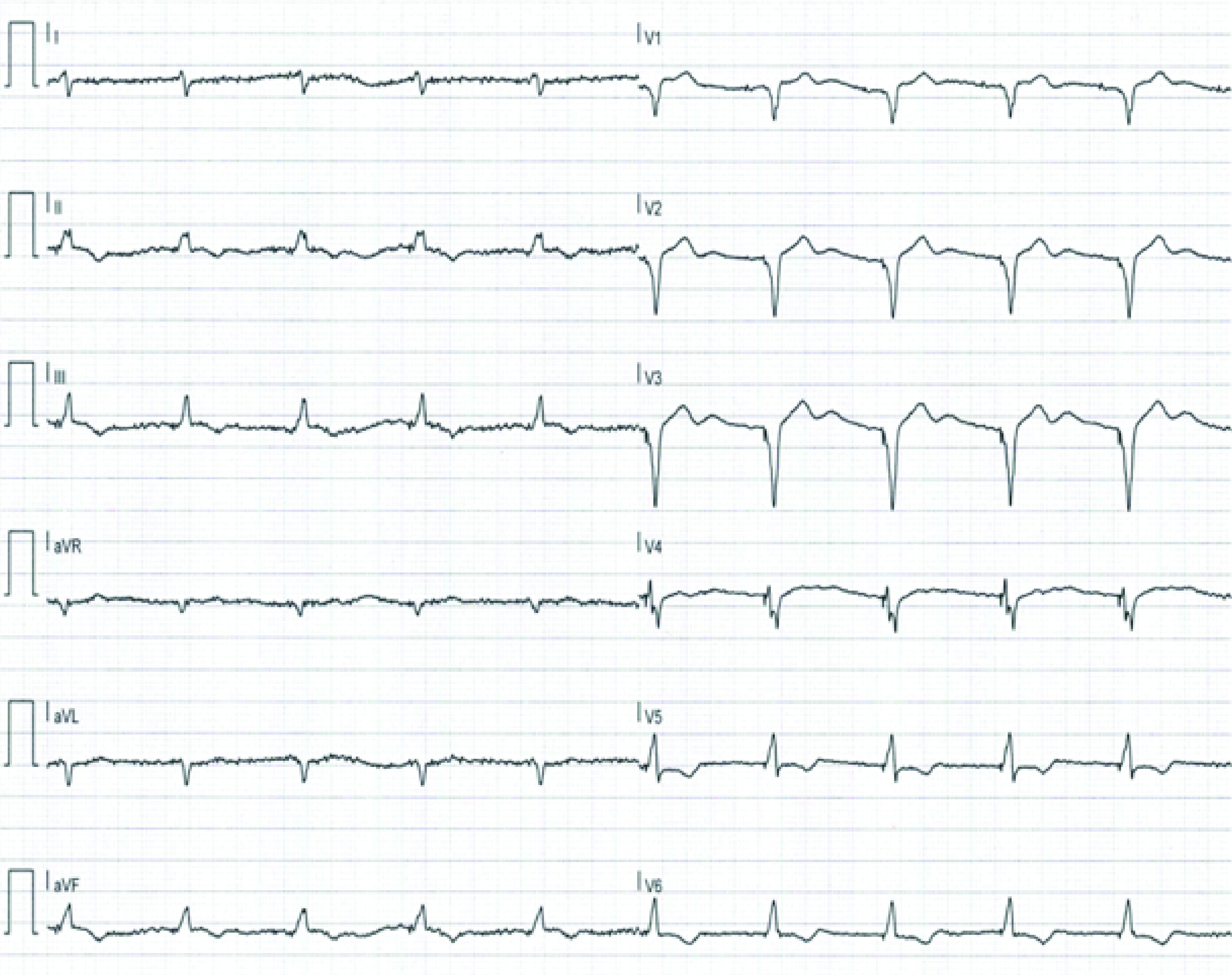
Pre-discharge ECG showing paced rhythm in dual-chamber pacing mode at 60 beats/min. In this figure, it is possible to appreciate the minor QRS duration compared with the pre-treatment ECG.

Device control performed at the end of the procedure showed optimal sensing and pacing parameters. A further nasopharyngeal swab for SARS-CoV-2 10 days later showed a negative result; the patient was discharged and 12-lead ECG was performed to assess the efficacy of biventricular stimulation on the duration of Q, R and S waves (Figure 4). At follow-up 1 month after implant, device control showed optimal sensing and pacing parameters, and the echocardiography documented an increase of EF calculated by the Simpson biplane method (35%). At 6-month follow-up, the device control showed regular parameters, with 99% biventricular stimulation and EF (calculated with the Simpson biplane method) increased to 42%. The biventricular stimulation, ensuring a synchronized contraction, improved LVEF and consequently the patient’s symptoms and quality of life. All healthcare professionals tested negative for SARS-CoV-2 at the subsequent nasopharyngeal swab.

## Discussion

We report a successful CRT-D implantation procedure in an 82-year-old man affected by COVID-19, recent MI, second-degree 2:1 AV block and LBBB.

At first, following the European Society for Cardiology recommendations [5], we tried a medical therapy approach with isoprenaline infusion while awaiting the recovery of the patient from COVID-19. However in patients with recent MI and second-degree 2:1 AV block, continuous infusion of isoprenaline is not the recommended therapy. Indeed, we immediately documented side effects of medical therapy such as frequent episodes of non-sustained ventricular tachycardia.

Given the duration of viral infection has been reported to be 21 days [6], we decided to perform the procedure of CRT-D implantation because of the presence of sinus rhythm with a QRS duration on ECG of ≥150 ms, LBBB QRS morphology and EF ≤35% [7]. The CRT, in patients with these characteristics, guarantees a reduction in risk of heart failure progression, ventricular tachyarrhythmias and death [3]. According to the Italian position paper, in COVID-19 patients, cephalic venous access is recommended compared with the subclavian route to avoid risk of pneumothorax [8]. Although cephalic access is a feasible method [9,10], it is challenging to position three leads. Thus we positioned the leads through the axillary vein, using an ultrasound-guided puncture, minimizing the risk of pneumothorax and other complications related to the blind subclavian puncture. We routinely use this technique and in our experience this approach is proven to be feasible, effective and safe, especially in COVID-19 patients, due to the lower risk of pneumothorax [11–13].

Furthermore, an antimicrobial envelope (Medtronic TYRX) was positioned in the pocket to reduce the bacterial infection risk, which was already high in this patient due to his comorbidities (i.e., diabetes), the ongoing inflammatory process caused by COVID-19 and the risk of multiresistant bacterial superinfection that unfortunately characterizes intensive care hospitalizations [14].

## Conclusion

The COVID-19 pandemic has seriously revolutionized the management of patients who need implantable cardioverter defibrillators, because the presence of infection leads to an unavoidable delay of implantation.

*De Vivo* *et al.* have already described an extraction of a cardiac implantable electronic device and a subsequent CRT-D implant in a patient with COVID-19 [15]. However, ours is the first report of a CRT-D *de novo* implant in a COVID-19 patient using an echo-guided axillary venous approach.

Although the implantation of CRT-D is quite widespread worldwide, it is a more complex and time-consuming procedure than the two-chamber cardiac implantable electronic device. In addition, the physicians’ clothing required during the pandemic made this procedure harder than a normal CRT implantation but, as we report, it was not impossible.

**Executive summary**

- We present a patient with COVID-19 affected by myocardial infarction, second-degree 2:1 atrioventricular block and left bundle branch block.
- COVID-19 infection has complicated the management of patients who need a cardiac implantable device.
- Despite the ongoing COVID-19 infection, we proceed to a cardiac resynchronization therapy defibrillator implantation, given the patient’s unstable cardiac rhythm.
- The procedure was performed using an echo-guided axillary venous approach to avoid complications of the implant, such as pneumothorax.
- During this pandemic period, our case report should encourage other physicians to make the right decision, avoiding any delay to life-saving procedures in COVID-19 patients.
